# Evolution of testicular architecture in the Drosophilidae: A role for sperm length

**DOI:** 10.1186/1471-2148-8-143

**Published:** 2008-05-13

**Authors:** Lukas Schärer, Jean-Luc Da Lage, Dominique Joly

**Affiliations:** 1 Division of Ultrastructural Research and Evolutionary Biology, Institute of Zoology, University of Innsbruck, Technikerstrasse 25, 6020 Innsbruck, Austria; 2 Laboratoire Evolution, Génomes et Spéciation, CNRS – UPR 9034, bat 13, Avenue de la Terrasse, 91198 Gif sur Yvette Cedex, France; 3 Evolutionary Biology, Zoological Institute, University of Basel, Vesalgasse 1, 4051, Basel, Switzerland

## Abstract

**Background:**

Evolutionary biologists have so far largely treated the testis as a black box with a certain size, a matching resource demand and a resulting sperm output. A better understanding of the way that the testis responds to selection may come from recent developments in theoretical biology aimed at understanding the factors that influence the evolution of tissue architecture (i.e. the logical organisation of a tissue). Here we perform a comparative analysis of aspects of testicular architecture of the fruit fly family Drosophilidae. Specifically, we collect published information on the number of first (or primary) spermatocytes in spermatogenesis, which allows to infer an important aspect of testicular architecture.

**Results:**

We show that testicular architecture is much more variable (both within and between species) than is generally appreciated. Moreover, the number of first spermatocytes is strongly correlated to the sperm length, which is inversely related to the sperm production, and thus the workload of the testis.

**Conclusion:**

Our study clearly documents that tissue architecture can evolve, and that in the Drosophilidae it may do so in response to sexual selection. We conclude that the testis of the Drosophilidae is a promising model organ to test recent models of tissue architecture.

## Background

It is generally accepted that sperm competition [1,2] can lead to selection for increased resource allocation towards the production of ejaculates and that different levels of sperm competition can cause rapid evolution of testis size [3-7]. However, selection due to sperm competition does not act on testis size per se, but on sperm production (sperm number and size). In other words, testis size evolves in response to the demand placed on sperm production by sperm competition. In spite of this, evolutionary biologists have to date largely treated the testis as a black box with a certain size, a matching resource demand and a resulting sperm output. Here we explore how the machinery of the testis may react to different sperm production demands (a change in which may be reflected in testis size).

**Figure 1:**
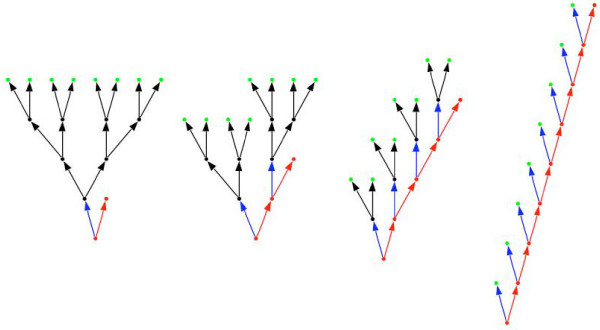
**Four different tissue architectures that lead to 8 differentiated cells (green) and one stem cell (red).** All tissue architectures require the same number of cell divisions, but individual cells divide different numbers of times. On the left the stem cell divides only once (*n*_s _= 1) to produce a transit cell (black) that in turn divides binomially three times (*n*_t _= 3), which produces a total of eight differentiated cells (*k *= *n*_s _× $2^{n_{\text{t}}}$ = 1 × 2^3 ^= 8). On the right the stem cell divides eight times and no transit cells are produced (*n*_s _= 8, *n*_t _= 0, *k *= 8 × 2^0 ^= 8). The other two tissue architectures are intermediate cases (centre left: *n*_s _= 2, *n*_t _= 2, *k *= 2 × 2^2 ^= 8; centre right, *n*_s _= 4, *n*_t _= 1, *k *= 4 × 2^1 ^= 8). The complete tissue will consist of *N *stem cells and thus be able to produce *T *= *N *× *k *differentiated cells. If the tissue is a testis each of these differentiated cells will go through the two meiotic divisions and will thus produce *T *= *N *× 4 × *k *sperm.

For this it is useful to consider recent theoretical models that investigate the factors that influence the evolution of optimal tissue architecture [8-10]. In this context the term tissue architecture refers to the logical organisation of a tissue (Fig. 1) rather than its detailed histological morphology. Although these theoretical models were originally formulated for tissues such as skin and gut epithelia they should also apply to the testis, which also has an epithelial organization.

The testicular epithelium is a complex tissue that contains different types of somatic and germ cells. In vertebrates somatic cells (e.g. Sertoli cells) make up an substantial portion of the epithelium and they have important nourishing functions for the developing sperm. In contrast, in many insects the somatic cells (e.g. cyst cells) contribute relatively little to the testis in terms of overall size. The germ cells are organized into long-lived basal stem cells (spermatogonia) and short-lived differentiating transit cells (spermatocytes and spermatids). The sperm can be produced through different patterns of stem vs. transit cell divisions (as in Fig. 1) and they are then shed at the surface of the testicular epithelium. The different division patterns can be seen to represent different tissue architectures.

Should we expect that selection on sperm production acts on tissue architecture? The main aspect that the theoretical models have investigated so far is how proliferation-induced mutations can affect the function of the tissue and the survival of the individual harbouring the tissue in the context of cancer. Epithelial tissues generally have high cell division rates and this can lead to a high risk of proliferation-induced somatic mutations. The question therefore is if certain tissue architectures may be less risk-prone than others. One theoretical study concludes that the architectural organization of a tissue into a 'linear process', with basal stem cells and differentiating transit cells, may itself be an adaptation to protect the tissue against the initiation of cancer [9]. Another study concludes that the workload of a tissue (i.e. the number of cells the tissue has to produce) can affect the optimal patterns of stem vs. transit cell divisions, and hence the optimal tissue architecture [8]. Finally, the division of the tissue into stem vs. transit cells may also result from constraints on the length of the transit cell lineage, which is expected to select for a lowered mutation rate in stem cells compared to transit cells [8,11].

Other factors that have not yet been modelled theoretically, but which we expect to be important are linked to the temporal and cellular demand that selection places on the tissue. A tissue that has to produce many cells in a short amount of time may need to shift cell divisions towards the transit cells in order to exploit the exponential nature of that part of the tissue architecture (i.e. the architecture on the left in Fig. 1 produces eight cells in four rounds of cell division, whereas the architecture on the right requires eight rounds of cell division). Moreover, in a short-lived or semelparous organism the fitness cost associated with developing testicular cancer may be lower than in a more long-lived iteroparous organism, where future reproduction is an important fitness component. Therefore more risk-averse division strategies are expected in iteroparous organisms. Finally, a tissue that has to produce large differentiated cells may reduce the number of transit cell divisions in order to avoid the halving of the cell size in every cell division.

As outlined above, sperm competition leads to frequent changes in the demand on sperm production imposed on the testis. Testicular architecture may therefore vary either within species or between closely related species. Moreover, because the demand on skin or gut epithelia is expected to be much less variable, the testis can serve as an interesting model tissue for studies of optimal tissue architecture. As we show below, the testis of the fruit fly family Drosophilidae allows to easily determine important aspects of testicular architecture and we therefore think it is a particularly promising model tissue to study its evolution. The first aim of our study is to investigate if variation exists in testicular architecture within and between closely related species among the Drosophilidae. The second aim is to attempt to explain at least some of the observed variation in tissue architecture, to discuss it in the context of the existing models, and to suggest directions for future research.

### The testis and spermatogenesis of the Drosophilidae

**Figure 2:**
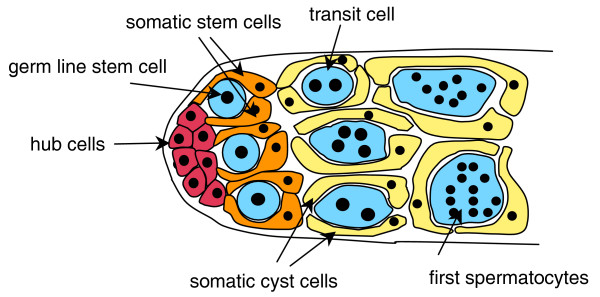
Organization of the testicular tip of *D. melanogaster *(modified from [30]).

The organization of the testis and the process of spermatogenesis are known in great detail for *Drosophila melanogaster *[12-14], and some other drosophilids [15-17], and they have features that greatly facilitate the inference of certain aspects of testicular architecture. The first step in spermatogenesis involves the division of a spermatogonial (germ line) stem cell and the division of two somatic stem cells (Fig. 2). During these divisions the mother cells remain attached to the somatic hub cells and the daughter cells together form a cyst in which the two somatic cyst cells jointly enclose the newly formed (transit) germ cell. The remaining spermatogenesis occurs within this cyst: the transit cell goes through several rounds of mitotic divisions leading to first spermatocytes (which are also frequently called primary spermatocytes), followed by the meiotic divisions leading to spermatids, and followed by spermiogenesis leading to differentiated sperm. The determination of the number of first spermatocytes (*F*) and spermatids (*S*) within a cyst therefore allows to accurately estimate the number of transit cell divisions *n*_t _[18-20]. If the transit cell divisions correspond to a perfectly bifurcating tree, we would as a general rule expect *F *= $2^{n_{\text{t}}}$ and *S *= 4 × $2^{n_{\text{t}}}$.

Another aspect for which there exists considerable knowledge is for sperm and testis size among members of the Drosophilidae. Sperm size is usually estimated as sperm length, which is highly variable within the family [21-23] and can reach truly gigantic dimensions [24-26]. Because sperm size trades off with sperm number [22] we can expect that the number of sperm produced per stem cell is smaller in species with large sperm. Sperm size may thus be important for testicular architecture. Furthermore, in many organisms testis size is expected to correlate with the total number of sperm produced by the testis, as larger testes will often have more stem cells. However, in the Drosophilidae this correlation appears unlikely. Although testis size is highly variable among different species of the Drosophilidae [21] this parameter is usually measured as testis length, which is tightly correlated to sperm length (relationship without phylogenetic correction, *r *= 0.94 [27]; relationship with phylogenetic correction, *r*^2 ^= 0.99 [28]). Within the Drosophilidae it is therefore unlikely that testis length provides much information on the number of stem cells in the testis. Only the direct observation of the testicular tip will allow to estimate this parameter [29,30], but there is little published information on this for drosophilids other than *D. melanogaster*.

In this study we assemble published data on the number of first spermatocytes and spermatids per cyst in different strains and species of the family Drosophilidae, and use these to estimate the number of transit cell divisions *n*_t_. We further assemble data on sperm size, which we expect may explain some of the variation in the number of sperm produced per stem cell, and which may thus be correlated to *n*_t_. We then perform a comparative analysis of independent contrasts using a molecular phylogeny and taxonomic information to correct for the phylogenetic relationships between the different species.

## Methods

### Literature data collection

We collected published information on a) the number of first (or primary) spermatocytes per cyst (i.e. the stage after all mitotic divisions, *F *= $2^{n_{\text{t}}}$), b) the number of spermatids per cyst (i.e. the stage after the two meiotic divisions have occurred, *S *= 4 × $2^{n_{\text{t}}}$), and c) sperm length (expected to be inversely correlated to the number of sperm produced per stem cell). We further add data on sperm length for two species, namely *Hirtodrosophila confusa *(n = 50 sperm) and *D. mercatorum *(n = 100 sperm). For species with heteromorphic sperm we used the length of the longer sperm morph in the analysis (thereby keeping the comparison within the fertilization-competent sperm morph, [31,32]). The data and references are listed in Additional file 1.

### Comparative analysis

As the backbone for the comparative analysis we used an *Amyrel*-based molecular phylogeny of the family Drosophilidae [33]. However, 14 species for which we had data were not represented in this molecular phylogeny. These were added using the taxonomical grouping into genus, group and subgroup (following the website Taxodros [34]), which in some cases led to polytomies in the proposed phylogeny. Moreover, one species, *D. suzukii*, was not added, because the molecular phylogeny suggested that the *suzukii *subgroup is polyphyletic (and it was therefore not clear to which subclade to add it). For the analysis of evolutionary relationships between the target variables we used CAIC 2.6.9[35] (available at [36]). For analyses the values for the number of first spermatocytes and sperm length were log-transformed. To investigate the relationship we used a linear regression forced through the origin, as suggested by the manual of CAIC.

## Results

### Variation in the number of first spermatocytes

**Figure 3:**
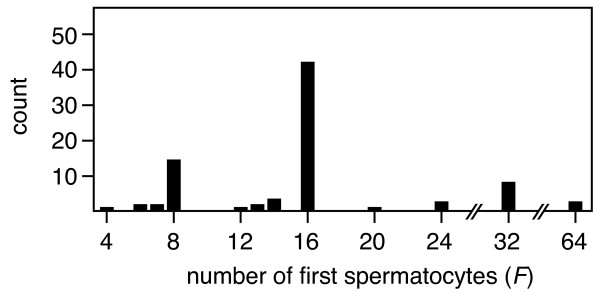
Frequency distribution of the number of first spermatocytes (*F*) per cyst in 100 species among the Drosophilidae.

**Figure 4:**
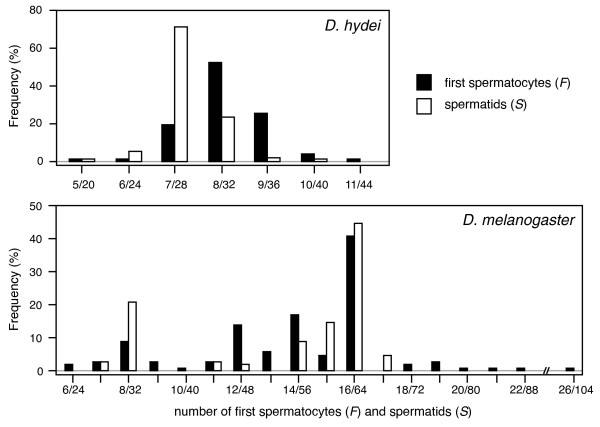
**Frequency distributions of the number of first spermatocytes (*F*) and spermatids (*S*) per cyst in *D. hydei *and *D. melanogaster *(redrawn from [19]).** Note a) that not all cysts contain the expected *F *= $2^{n_{\text{t}}}$ spermatocytes, but that *F *can deviate both above and below the expected values by small increments, b) that the number of spermatids per cyst (*S*) approximately reflects the patterns in *F*, but that the mode of the two distributions can be somewhat shifted relative to each other, and c) that in *D. melanogaster *both the *F *and *S *distributions are clearly bimodal.

The published literature yields data on the number of first spermatocytes for 100 species among the Drosophilidae (Additional file 1), and suggests that the patterns of cell division during spermatogenesis are a) very variable within the family (spanning about one order of magnitude), and b) much more variable than the general rule of *F *= $2^{n_{\text{t}}}$ would suggest (Fig. 3). Four independent research groups report numbers of first spermatocytes (and also spermatids per cyst) that deviate from this rule. A Japanese research group screened 78 species within the Drosophilidae, and found a general agreement with the expected patterns, namely 8, 16, 32, or 64 first spermatocytes produced per cyst in most species [18]. However, several species deviated consistently from this pattern, producing intermediate numbers of first spermatocytes. Next, a German research group documented extensive variation, not only within species, but also within individuals of a species [16,19,37]. In Figure 4 we redraw published distributions from *D. hydei *and *D. melanogaster *from [19], which show the kind of variation in the number of first spermatocytes and spermatids per cyst, and the strikingly broad and bimodal distribution in *D. melanogaster*. Later the Japanese research group confirmed and extended such observations [20,38], and identified variation in species that they had initially reported to fit the expected pattern. Moreover, they were able to document significant variation in the number of first spermatocytes and spermatids per cyst both within and between different isofemale lines of *D. virilis *[39]. More importantly, they showed that crosses between two of those isofemale lines yielded an intermediate phenotype, which clearly suggests that heritable genetic variation underlies these traits [40]. It therefore appears likely that testicular architecture can respond to selection. Finally, recent papers by two other groups also report and/or confirm deviations the *F *= $2^{n_{\text{t}}}$ rule for several species [28,41].

It is thus clear that these deviations are widespread and it is important to note that small to intermediate levels of variation around, and deviations from, the *F *= $2^{n_{\text{t}}}$ pattern also occur in species that were initially classified as fitting the expected pattern, such as in *D. melanogaster *or *D. simulans *[18]. This suggests that there is much more variation in the number of first spermatocytes than is generally appreciated.

### Evolution of the number of first spermatocytes

**Figure 5:**
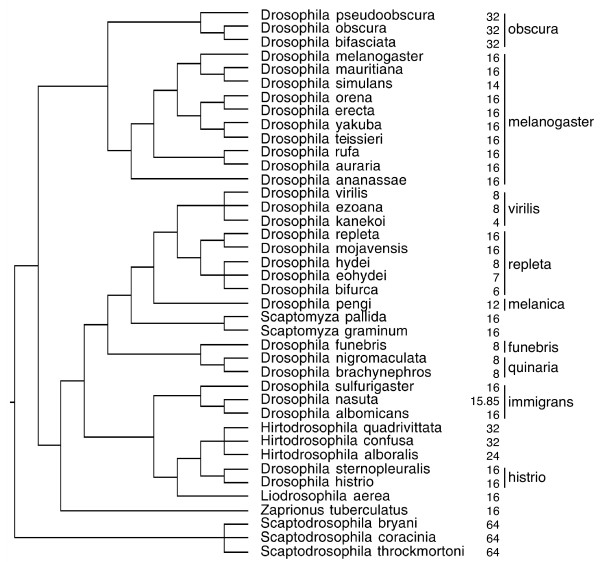
**Phylogenetic relationships for the members of the Drosophilidae that were included in the comparative analysis of independent contrasts (see also Figure 6B), the number of first spermatocytes per cyst in those species, and the species-groups for the species within the genus *Drosophila*.** Note that a) the same number of first spermatocytes per cyst can be found in several unrelated groups and b) that the number of first spermatocytes per cyst can vary within a species-group.

**Figure 6:**
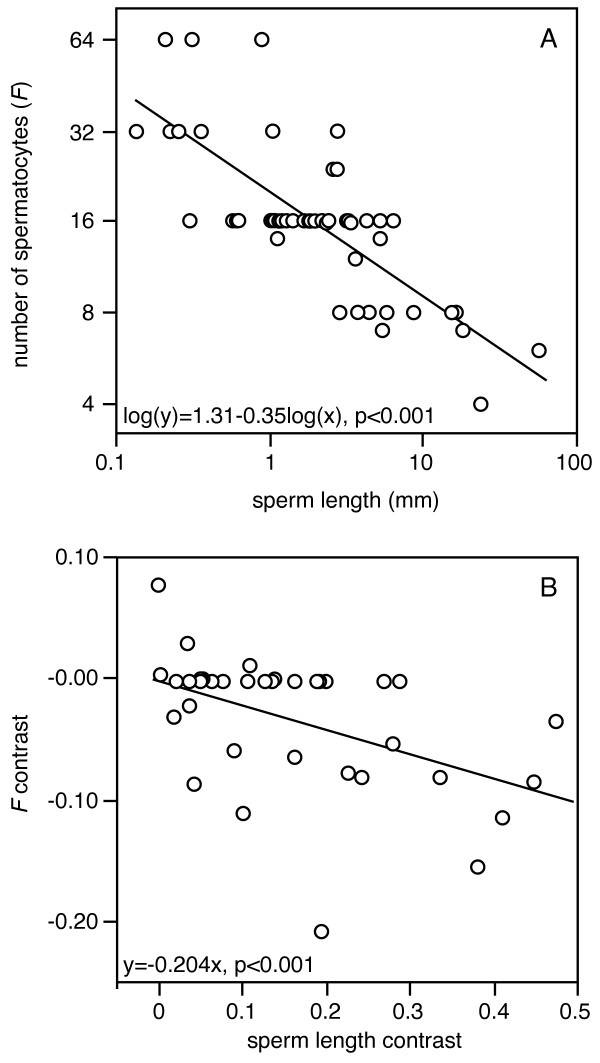
**Relationships between sperm length and the number of first spermatocytes (*F*) per cyst: a) the relationship for the 57 species for which we have values of both sperm length and *F *(see Additional file**1** for the data and references)(linear regression, *F*_1,55 _= 86.4, *p *< 0.001), and b) the phylogenetically controlled relationship from the comparative analysis for the 40 species listed in Figure 5 (independent contrasts, linear regression forced through the origin, *F*_1,34 _= 30.2, *p *< 0.001).**

Most species that we were able to include in the comparative analysis appear to approximately fit the *F *= $2^{n_{\text{t}}}$ pattern, but several species show intermediate patterns or slight deviations (Fig. 5). Considerable variation occurs within some taxonomic groups (e.g. see the values for the *virilis *and *repleta *species groups), whereas other groups seem less variable (e.g. the *melanogaster *species group). Overall the number of first spermatocytes significantly co-varies with sperm length (Fig. 6A for the relationship without phylogenetic correction). The comparative analysis of independent contrasts shows that there is a significant evolutionary covariance between sperm length and the number of first spermatocytes, which is independent of the phylogenetic relationships between the species (Fig. 6B). Although there appears to be considerable phylogenetic inertia over part of the distribution (as evidenced by the large number of zeroes for the first spermatocyte contrast), the highest sperm length contrasts are associated with a reduced number of first spermatocytes per cyst.

## Discussion

### Variation in the number of first spermatocytes

Our study clearly suggests that the number of first spermatocytes does not always fit the expected *F *= $2^{n_{\text{t}}}$ pattern (Figs. 3 and 4). Two types of deviation are evident. On one hand there is large inter-specific variation in *F *(ranging from 4 to 64 first spermatocytes), with some species having values that are consistently intermediate between two $2^{n_{\text{t}}}$ levels (e.g. *D. bifurca*, *F *= 6; *D. pengi*, *F *= 12; *Hirtodrosophila alboralis *and *D. curviceps*, *F *= 24; see also Fig. 3 and 6A).

On the other hand there is considerable intra-specific variation in *F *(Fig. 4 and Additional file 1). Surprisingly, the most striking pattern occurs in the best studied species, *D. melanogaster*, which shows a very broad bimodal distribution with clear modes at 8 and 16 first spermatocytes (Fig. 4). And although this was reported over 20 years ago [19], it is not generally appreciated. Almost all major reviews on *D. melanogaster *we consulted state that spermatogenesis occurs in a completely synchronous way and always leads to *F *= 2^4 ^= 16 first spermatocytes and *S *= 4 × 2^4 ^= 64 sperm per cyst [12,13,42,43]. Only one older review [14] briefly mentions some evidence for 32 instead of 64 spermatids per cyst in *D. melanogaster*. And while the distribution in *D. melanogaster *is particularly striking and maybe unique in its bimodality, there are many species that have distributions of the type and magnitude of *D. hydei*, which we here show as a representative example (Fig. 4). In fact, most species that were studied in detail, show at least some level of variation (Additional file 1 gives more detailed information on this variation).

Some of the data we report were collected using an *in vitro *system in which the germ cells undergo partial spermatogenesis [17]. So deviations from the *F *= $2^{n_{\text{t}}}$ pattern could in these cases potentially indicate *in vitro *artefacts. However, the data for spermatids were collected based on cross-sections of resin-embedded testes extracted from adult males, and thus are independent of such potential artefacts. The good fits between the distributions for first spermatocytes and spermatids (Fig. 4) suggest that the deviations from the *F *= $2^{n_{\text{t}}}$ pattern are real. This clearly suggests that, contrary to the general assumption, cell divisions do not have to be completely synchronized within a cyst.

Interestingly, based on a number of published images of cysts containing first spermatocytes it appears that all cells have the same size, even if the particular strain or species has intermediate numbers of first spermatocytes (e.g. *F *= 6 in *D. bifurca *in Fig 1B of [19] or *F *= 9 in strain A12 of *D. virilis *in Fig 1C of [39]). This observation is surprising if some cells go through fewer or more rounds of cell division than other cells in a cyst. We suggest that the function of the well known phenomenon of cytoplasmic bridges between developing spermatocytes and spermatids may be to allow equalization of the cytoplasm following asynchronous cell division. We thereby add to a growing list of hypotheses for the function of cytoplasmic bridges, such as assuring synchronous cell division, nutrient transport, or avoiding conflict between the father and its sperm (reviewed in [44,45]).

### Evolution of the number of first spermatocytes

One important prerequisite for the evolution of tissue architecture is that genetic variation exists for traits that determine the architecture. The general assumption in most species is that the number of first spermatocytes is fixed and species-specific. If that were the case it is hard to imagine how this trait could evolve, because there would be no heritable variation on which selection could act. Our results clearly suggest that the number of first spermatocytes *F *is variable within species and that it does evolve within the Drosophilidae. However, it is also evident that the data show a strong stratification into the expected levels of *F *= $2^{n_{\text{t}}}$ (primarily 8,16, or 32 first spermatocytes, see Fig. 3 and 6A) and there appears to be considerable phylogenetic inertia, as several species groups show none or little variation around one of these levels (exceptions are the *virilis *and *repleta *groups, which span two and more than two levels respectively). However, the current analysis only considers the average or modal *F *values for the different species and ignores the sometimes considerable intra-specific variation. We suspect that more detailed analyses would reveal further variation in species that are currently considered to accurately fit the *F *= $2^{n_{\text{t}}}$ pattern.

### Evidence for a role of sperm competition

The extensive variation in the number of first spermatocytes we report is strongly negatively correlated with sperm length, which in turn is strongly negatively correlated with the number of sperm produced [22]. This suggests that the testicular architecture shifts towards the transit cell lineage in the species that produce many small sperm. So can we conclude that sperm competition plays a role in this shift? Little is known about the mating system and the intensity of sperm competition in the majority of species we included in our study, and it is still not entirely clear what the function of the giant sperm is. However, it is currently thought that the evolution of sperm length is linked to sexual conflict over the usage of sperm [46,47], and that it thus represents the outcome of postcopulatory sexual selection, or more specifically of a complex interaction between sperm competition and cryptic female choice. It therefore appears possible that sexual selection can influence testicular architecture, but whether it is due to selection on sperm size or sperm number remains to be tested.

Although one of the earliest comparative studies on the evolution of testis size [3] reported a potential influence of sperm competition on the organization of the testis (i.e. the ratio of spermatogenic tissue to interstitial tissue) very little data has been collected on such aspects since. We are aware of only one other study that has looked at the morphological organization of the testis from a sperm competition perspective. This comparative study in primates showed considerable variation in spermatogenic efficiency (i.e. spermatid production per unit of testicular tissue), but this variation was not related to differences in the mating system between the studied species [48].

### Experimental approaches to study testicular architecture

An interesting avenue for future research would be to experimentally test the evolvability of testicular architecture by artificially selecting either on the number of first spermatocytes or on sperm production. Given the variation we describe it is clear that artificial selection on testis size per se may lead to a different response than selection on sperm production. Another interesting approach could be the recent experimental evolution experiment in which *D. melanogaster *was grown for many generations under different levels of sexual selection (i.e. monogamy vs. polygamy) [49]. This regime appears to have led to a reduced sperm production by males held under monogamy [50]. So flies from these (or similar) lines could be used to evaluate if *n*_t _or other parameters of testicular architecture have evolved in response to the selection regime.

Instead of investigating evolutionary responses one could also check if there is any phenotypic plasticity in testicular architecture. One study mentions unpublished results that suggest that different food levels and temperatures have no effect on the number of first spermatocytes [40]), suggesting that it is not phenotypically plastic in response to these environmental variables. Another study showed that sperm length varies under different temperature constraints [51], but that study did not investigate variation in the number of first spermatocytes per cyst.

Phenotypic plasticity in male allocation has been reported in a number of organisms in response to environmental cues that indicate future sperm competition risk [52-55]. Such variation in male allocation may lead to different numbers of sperm produced, and it could therefore be interesting to test if it also leads to changes in testicular architecture.

### Comparative approaches to study testicular architecture

As outlined above our comparative analysis reveals a highly significant association between the number of first spermatocytes and sperm length. Given that we expect that sperm length (*SL*) is inversely related to the total number of sperm (*T*) produced by a fly [22], we can suggest that at least part of the variation in *T *is explained by variation in *n*_t _because 1/*SL *≈ *T *= *N *× *k *= *N *× *n*_s _× (2^*n *^_t_)^2^. However, it appears of course possible that the number of stem cells (*N*) and the number of stem cell divisions (*n*_s_) also vary between species, and these will therefore also have to be estimated for a more complete comparative analysis. We think that it is possible to do this for the testis of the Drosophilidae, and that this tissue therefore is an ideal model organ to test the existing tissue architecture models [8,10,56]. In Additional file 2 we outline what data should be collected to more fully parameterize testicular architecture. Moreover, it would be highly relevant to collect more comparative data on the variation in mating systems of the different drosophilid species. The recent sequencing of the genomes of 12 *Drosophila *species covering a large fraction of the genus *Drosophila *should facilitate the establishment of microsatellite markers that could be used on many species within this genus (or maybe even within the entire Drosophilidae). Such markers would make it much easier to obtain comparative data on levels of multiple paternity for a range of species.

## Conclusion

Evolutionary biologists have generally treated the testis as a black box which simply has a size that indicates its resource use (or reproductive allocation), and a resulting sperm production that is tailored to the mating system requirements. So the prevailing idea is that the testis simply responds to varying demands by changing its size. However, as a consequence of variation in the strength of sexual selection the testis not only has to produce drastically different numbers of cells, but also cells of highly variable morphology and complexity. This means that there are few tissues which are under more variable and rapidly changing selection pressures than the testis. By investigating the way in which this organ responds to these selection pressures, either experimentally or based on comparative testicular architecture, we can expect to learn a lot about the evolutionary importance of tissue architecture.

## Authors' contributions

LS conceived the idea to use the testis as a model organ to study tissue architecture and DJ contributed the expertise about drosophilid spermatogenesis. LS and DJ assembled the comparative data set during a research visit in Gif sur Yvette. J-LdL provided phylogenetic advice and access to a then still unpublished molecular phylogeny. LS and DJ analysed the data and drafted the manuscript during a research visit in Innsbruck. All authors have critically read and approved the final manuscript.

## Supplementary Material

Additional file 1 Distribution of the number of first spermatocytes and spermatids per cyst, and sperm length among 100 members of the family Drosophilidae. Click here for file

Additional file 2 Parameterization of the testicular architecture of the Drosophilide. Click here for file
