# Supplementary material for: Evolution of testicular architecture in the Drosophilidae: A role for sperm length
Source: BMC Evol Biol. 2008 May 13;8:143. doi: 10.1186/1471-2148-8-143 (PMC2396631; doi:10.1186/1471-2148-8-143)
Supplement: Additional file 1 — Distribution of the number of first spermatocytes and spermatids per cyst, and sperm length among 100 members of the family Drosophilidae. [file 1471-2148-8-143-S1.doc]

Additional file 1: Distribution of the number of first spermatocytes (*F*) and spermatids (*S*) per cyst, and sperm length (*SL*) among 100 members of the family Drosophilidae. Genera, groups, subgroups and species are listed in alphabetical order. Species marked by an asterisk (*) were included in the Figure 6A. Values for *F* marked with (†) represent the mode of the distribution. R1 is the reference for *F* and *S* and R2 the reference for *SL* (‡ indicates values measured in this study) Comments give more detailed information on *F* and *S* from the main reference and, where available, relevant additional references for the species.

| Genus | Group | Subgroup | Species | *F* | *S* | R_1_ | *SL* | R_2_ | Comments |
| --- | --- | --- | --- | --- | --- | --- | --- | --- | --- |
| *Amiota* |  |  | *dispina** | 16 |  | [1] | 0.64 | [2] |  |
| *Chymomyza* |  |  | *procnemis* | 16 |  | [1] |  |  |  |
| *Dichaetophora* |  |  | *tenuicauda* | 16 |  | [1] |  |  | was listed as *Drosophila* |
| *Drosophila* |  |  | *maculinotata* | 24 |  | [1] |  |  |  |
| *Drosophila* | *acutissima* |  | *acutissima* | 16 |  | [1] |  |  |  |
| *Drosophila* | *bizonata* |  | *bizonata* | 16 |  | [1] |  |  |  |
| *Drosophila* | *busckii* |  | *busckii** | 16 |  | [1] | 1.1 | [3] |  |
| *Drosophila* | *funebris* |  | *multispina* | 8 |  | [1] |  |  |  |
| *Drosophila* | *funebris* | *funebris* | *funebris** | 8 |  | [1] | 8.85 | [4] |  |
| *Drosophila* | *funebris* | *macrospina* | *macrospina* | 8 |  | [1] |  |  |  |
| *Drosophila* | *funebris* | *macrospina* | *subfunebris* | 8 |  | [1] |  |  |  |
| *Drosophila* | *histrio* |  | *histrio** | 16 |  | [1] | 3.23 | [3] | slight variation in *F* |
| *Drosophila* | *histrio* |  | *sternopleuralis** | 16 |  | [1] | 1.29 | [3] |  |
| *Drosophila* | *immigrans* | *curviceps* | *curviceps** | 24.02 |  | [5] | 2.6 | [3] | *F=*19-29, mode at 24, flat distribution, see also [1] |
| *Drosophila* | *immigrans* | *hypocausta* | *hypocausta* | 16 |  | [1] |  |  |  |
| *Drosophila* | *immigrans* | *hypocausta* | *neohypocausta* | 15.78 |  | [5] |  |  | *F=*13-16, mode at 16 |
| *Drosophila* | *immigrans* | *immigrans* | *formosana* | 15.7 |  | [5] |  |  | *F=*12-17, mode at 16 |
| *Drosophila* | *immigrans* | *immigrans* | *immigrans* | 14 | 64 | [6] |  |  | *F=*7-17, mode at 11 or 14, S=(52, 56, 60, 64, 68), odd distribution, see also [1, 5, 7] |
| *Drosophila* | *immigrans* | *nasuta* | *albomicans** | 16 |  | [1] | 4.33 | [8] |  |
| *Drosophila* | *immigrans* | *nasuta* | *kohkoa** | 15.79 |  | [5] | 2.41 | [8] | *F=*12-16, mode at 16 |
| *Drosophila* | *immigrans* | *nasuta* | *nasuta** | 15.85 |  | [5] | 3.42 | [8] | *F=*12-16, mode at 16 |
| *Drosophila* | *immigrans* | *nasuta* | *sulfurigaster** | 16 |  | [1] | 1.99 | [4] | see also [5] for *D. s. albostrigata* (mean *F=*15.86, range 12-16, mode at 16) |
| *Drosophila* | *immigrans* | *quadrilineata* | *annulipes* | 20.41 |  | [5] |  |  | *F=*14-27, mode at 20, flat distribution, see also [1] |
| *Drosophila* | *immigrans* | *quadrilineata* | *quadrilineata* | 15.88 |  | [5] |  |  | *F=*15-16, mode at 16, see also [1] |
| *Drosophila* | *melanica* |  | *melanica* | 8 |  | [9] |  |  |  |
| *Drosophila* | *melanica* |  | *micromelanica* | 8 |  | [9] |  |  |  |
| *Drosophila* | *melanica* |  | *moriwakii* | 16 |  | [1] |  |  |  |
| *Drosophila* | *melanica* |  | *pengi** | 12 |  | [1] | 3.71 | [2] | slight variation in *F* |
| *Drosophila* | *melanogaster* | *ananassae* | *ananassae** | 16 |  | [1] | 3.33 | [4] |  |
| *Drosophila* | *melanogaster* | *ananassae* | *bipectinata* | 16 |  | [1] |  |  |  |
| *Drosophila* | *melanogaster* | *ficusphila* | *ficusphila** | 16 |  | [1] | 1.84 | [2] |  |
| *Drosophila* | *melanogaster* | *melanogaster* | *erecta** | 16 |  | [10] | 1.21 | [4] | *F=*15-17 |
| *Drosophila* | *melanogaster* | *melanogaster* | *mauritiana** | 16 |  | [10] | 1.04 | [4] | *F=*8-16 |
| *Drosophila* | *melanogaster* | *melanogaster* | *melanogaster** | 16† | 64 | [6] | 1.90 | [4] | *F=*6-26, S=(28, 32, 44, 48, 56, 60, 64, 68), bimodal, used higher peak, see also [1, 7, 11] |
| *Drosophila* | *melanogaster* | *melanogaster* | *orena** | 16 |  | [10] | 1.44 | [4] | *F=*15-17 |
| *Drosophila* | *melanogaster* | *melanogaster* | *simulans** | 14† | 64 | [6] | 1.12 | [4] | *F=*10-16, S=(56, 60, 64), odd distribution, see also [1, 7, 10] |
| *Drosophila* | *melanogaster* | *melanogaster* | *teissieri** | 16 |  | [10] | 1.84 | [4] | *F=*8-18 |
| *Drosophila* | *melanogaster* | *melanogaster* | *yakuba** | 16 |  | [10] | 1.68 | [4] |  |
| *Drosophila* | *melanogaster* | *montium* | *auraria** | 16 |  | [1] | 2.22 | [2] |  |
| *Drosophila* | *melanogaster* | *montium* | *kikkawai* | 16 |  | [1] |  |  |  |
| *Drosophila* | *melanogaster* | *montium* | *pectinifera* | 16 |  | [1] |  |  |  |
| *Drosophila* | *melanogaster* | *montium* | *punjabiensis* | 16 |  | [1] |  |  |  |
| *Drosophila* | *melanogaster* | *montium* | *rufa** | 16 |  | [1] | 5.37 | [2] |  |
| *Drosophila* | *melanogaster* | *suzukii* | *pulchrella* | 16 |  | [1] |  |  |  |
| *Drosophila* | *melanogaster* | *suzukii* | *suzukii** | 16 |  | [1] | 2.22 | [2] |  |
| *Drosophila* | *melanogaster* | *takahashii* | *lutea* | 16 |  | [1] |  |  |  |
| *Drosophila* | *melanogaster* | *takahashii* | *takahashii* | 16 |  | [1] |  |  |  |
| *Drosophila* | *nannoptera* |  | *acanthoptera** | 8 |  | [9] | 5.83 | [12] |  |
| *Drosophila* | *nannoptera* |  | *nannoptera** | 8 |  | [9] | 15.74 | [12] |  |
| *Drosophila* | *nannoptera* |  | *pachea** | 8 |  | [9] | 16.53 | [12] |  |
| *Drosophila* | *nannoptera* |  | *wassermanni** | 8 |  | [9] | 4.52 | [12] |  |
| *Drosophila* | *obscura* | *obscura* | *bifasciata** | 32 |  | [1] | 0.23 | [13] |  |
| *Drosophila* | *obscura* | *obscura* | *imaii* | 32 |  | [1] |  |  |  |
| *Drosophila* | *obscura* | *obscura* | *obscura** | 32 |  | [1] | 0.14 | [13] |  |
| *Drosophila* | *obscura* | *pseudoobscura* | *miranda* | 32 |  | [1] |  |  | see also [14] |
| *Drosophila* | *obscura* | *pseudoobscura* | *pseudoobscura** | 32 | 128 | [1] | 0.26 | [13] | value of S from [11], see also [14] |
| *Drosophila* | *polychaeta* |  | *daruma* | 16 |  | [1] |  |  |  |
| *Drosophila* | *quinaria* |  | *brachynephros** | 8 |  | [1] | 2.87 | [2] |  |
| *Drosophila* | *quinaria* |  | *kuntzei* | 8 |  | [1] |  |  |  |
| *Drosophila* | *quinaria* |  | *nigromaculata** | 8 |  | [1] | 3.85 | [2] |  |
| *Drosophila* | *repleta* | *hydei* | *bifurca** | 6† | 20 | [6] | 58.36 | [15] | *F=*4-7, S=(16, 20, 24), see also [7, 9, 16] |
| *Drosophila* | *repleta* | *hydei* | *eohydei** | 7† | 28 | [6] | 18.11 | [9] | *F=*5-8, S=(24, 28), see also [9] |
| *Drosophila* | *repleta* | *hydei* | *hydei** | 8† | 28 | [6] | 16.90 | [4] | *F=*5-11, S=(20, 24, 28, 32, 36, 40) , see also [1, 7, 9, 16, 17] |
| *Drosophila* | *repleta* | *hydei* | *neohydei* | 6 | 20 | [7] |  |  |  |
| *Drosophila* | *repleta* | *melanopalpa* | *melanopalpa* | 13 | 52 | [6] |  |  | *F=*9-16, S=44-60, used median |
| *Drosophila* | *repleta* | *mercatorum* | *mercatorum** | 16 |  | [1] | 1.16 | ‡ |  |
| *Drosophila* | *repleta* | *mulleri* | *mojavensis** | 16 | 64 | [9] | 1.9 | [9] |  |
| *Drosophila* | *repleta* | *mulleri* | *stalkeri* | 16 |  | [1] |  |  |  |
| *Drosophila* | *repleta* | *repleta* | *fulvimacula* | 13 | 52 | [7] |  |  | see also [16], *F=*9-14 |
| *Drosophila* | *repleta* | *repleta* | *repleta** | 16 |  | [1] | 6.55 | [4] |  |
| *Drosophila* | *robusta* | *lacertosa* | *lacertosa** | 7 |  | [1] | 5.44 | [2] | slight variation in *F* |
| *Drosophila* | *robusta* | *robusta* | *sordidula** | 14 |  | [1] | 5.24 | [2] |  |
| *Drosophila* | *testacea* |  | *testacea* | 16 |  | [1] |  |  |  |
| *Drosophila* | *tumiditarsus* |  | *tumiditarsus* | 14 |  | [1] |  |  |  |
| *Drosophila* | *virilis* |  | *ezoana** | 8 |  | [1] | 15.33 | [18] |  |
| *Drosophila* | *virilis* |  | *kanekoi** | 4 |  | [19] | 24.29 | [18] | *F=*2-6, data from isofemale lines suggest line differences |
| *Drosophila* | *virilis* |  | *virilis** | 8 | 32 | [6] | 4.56 | [4] | *F=*5-16, S=(28, 32, 36, 40), see also [1, 7, 20] |
| *Hirtodrosophila* | *melanderi* |  | *makinoi* | 16 |  | [1] |  |  |  |
| *Hirtodrosophila* | *quadrivittata* |  | *alboralis** | 24 |  | [1] | 2.78 | [2] | was listed as *Drosophila* |
| *Hirtodrosophila* | *quadrivittata* |  | *sexvittata* | 8 |  | [1] |  |  | was listed as *Drosophila* |
| *Hirtodrosophila* | *quadrivittata* | *confusa* | *confusa** | 32 |  | [1] | 2.77 | ‡ | was listed as *Drosophila* |
| *Hirtodrosophila* | *quadrivittata* | *quadrivittata* | *fascipennis* | 32 |  | [1] |  |  | was listed as *Drosophila* |
| *Hirtodrosophila* | *quadrivittata* | *quadrivittata* | *quadrivittata** | 32 |  | [1] | 0.36 | [2] | was listed as *Drosophila* |
| *Leucophenga* |  |  | *maculata** | 16 |  | [1] | 1.08 | [2] |  |
| *Leucophenga* |  |  | *magnipalpis** | 16 |  | [1] | 1.16 | [2] |  |
| *Leucophenga* |  |  | *ornatipennis* | 16 |  | [1] |  |  |  |
| *Liodrosophila* |  |  | *aerea** | 16 |  | [1] | 2.47 | [2] |  |
| *Lordiphosa* |  |  | *denticeps* | 8 |  | [1] |  |  |  |
| *Microdrosophila* |  |  | *cristata* | 32 |  | [1] |  |  |  |
| *Microdrosophila* |  |  | *purpurata** | 32 |  | [1] | 1.06 | [2] |  |
| *Paraleucophenga* |  |  | *argentosa* | 16 |  | [1] |  |  |  |
| *Phortica* |  |  | *variegata** | 16 |  | [1] | 0.63 | [2] |  |
| *Scaptodrosophila* |  |  | *coracinia** | 64 |  | [1] | 0.21 | [2] |  |
| *Scaptodrosophila* |  |  | *throckmortoni** | 64 |  | [1] | 0.32 | [2] | was listed as *Drosophila* |
| *Scaptodrosophila* | *bryani* |  | *bryani** | 64 |  | [1] | 0.9 | [2] |  |
| *Scaptomyza* |  |  | *consimilis* | 16 |  | [1] |  |  |  |
| *Scaptomyza* |  |  | *graminum** | 16 |  | [1] | 0.58 | [2] |  |
| *Scaptomyza* |  |  | *pallida** | 16 |  | [1] | 0.31 | [2] | was listed as *Parascaptomyza* |
| *Zaprionus* |  |  | *tuberculatus** | 16 |  | [1] | 3.31 | [4] |  |
| *Zaprionus* |  |  | *vittiger* | 16 |  | [1] |  |  |  |

References:

1. Kurokawa H, Hihara F: **Number of first spermatocytes in relation to phylogeny of *Drosophila* (Diptera: Drosophilidae)**. *Int J Insect Morphol Embryol* 1976, **5**(1):51-63.

2. Hihara F, Kurokawa H: **The sperm length and the internal reproductive organs of *Drosophila* with special references to phylogenetic relationships**. *Zool Sci* 1987, **4**(1):167-174.

3. Joly D, Bressac C, Devaux J, Lachaise D: **Sperm length diversity in Drosophilidae**. *Dros Inf Serv* 1991, **70**:104-108.

4. Joly D, Bressac C: **Sperm length in Drosophilidae (Diptera): estimation by testis and receptacle lengths**. *Int J Insect Morphol Embryol* 1994, **23**(2):85-92.

5. Oguma Y, Kurokawa H, Kusama T: **Number of primary spermatocytes in the *Drosophila immigrans* (Sturtevant) group (Diptera: Drosophilidae)**. *Int J Insect Morphol Embryol* 1987, **16**(1):85-89.

6. Liebrich W, Hanna PJ, Hess O: **Evidence for asynchronous mitotic cell divisions in secondary spermatogonia of *Drosophila***. *Int J Invertebr Reprod* 1982, **5**(6):305-310.

7. Hanna PJ, Liebrich W, Hess O: **Spermatocytes in *Drosophila* not appearing to be produced by synchronous divisions of definitive spermatogonia**. *Dros Inf Serv* 1982, **58**:72-73.

8. Hatsumi M, Wakahama KI: **The sperm length and the testis length in *Drosophila nasuta* subgroup**. *Jpn J Genetics* 1986, **61**(3):241-244.

9. Pitnick S: **Investment in testes and the cost of making long sperm in *Drosophila***. *Am Nat* 1996, **148**(1):57-80.

10. Joly D: **Number of spermatocytes per cyst in six species of the *Drosophila melanogaster* subgroup**. *Dros Inf Serv* 1994, **75**:115.

11. Lindsley DL, Tokuyasu KT: **Spermatogenesis**. In: *The Genetics and Biology of Drosophila.* Edited by Ashburner M, Wright TRF, vol. 2. London: Academic Press; 1980: 225-294.

12. Pitnick S, Markow TA: **Male gametic strategies: sperm size, testes size, and the allocation of ejaculate among successive mates by the sperm-limited fly *Drosophila pachea* and its relatives**. *Am Nat* 1994, **143**(5):785-819.

13. Joly D, Cariou ML, Lachaise D, David JR: **Variation of sperm length and heteromorphism in drosophilid species**. *Genet Sel Evol* 1989, **21**(3):283-293.

14. Dobzhansky T: **Studies on hybrid sterility. I. Spermatocytes in pure and hybrid *D. pseudoobscura***. *Z Zellforsch mikrosk Anat* 1934, **21**:169-223.

15. Joly D, Bressac C, Lachaise D: **Disentangling giant sperm**. *Nature* 1995, **377**(6546):202.

16. Hanna PJ, Liebrich W, Hess O: **Evidence against a (2)^n^ synchronous increase of spermatogonia to produce spermatocytes in *Drosophila hydei***. *Gamete Res* 1982, **6**(4):365-370.

17. Meyer GF: **Spermiogenese in normalen und Y-defizienten Männchen von *Drosophila melanogaster* und *D. hydei***. *Z Zellforsch mikrosk Anat* 1967, **84**(2):141-175.

18. Pitnick S, Markow TA, Spicer GS: **Delayed male maturity is a cost of producing large sperm in *Drosophila***. *Proc Natl Acad Sci USA* 1995, **92**(23):10614-10618.

19. Oguma Y, Kurokawa H: **The least cell number of first spermatocytes per cyst found in *Drosophila kanekoi***. *Jpn J Genetics* 1984, **59**(3):263-265.

20. Oguma Y, Kurokawa H, Sogawa Y: **Variation of the number of first spermatocytes in relation to fertility in *Drosophila virilis***. *Jpn J Genetics* 1984, **59**(6):565-575.
