# Supplementary material for: Evolution of testicular architecture in the Drosophilidae: A role for sperm length
Source: BMC Evol Biol. 2008 May 13;8:143. doi: 10.1186/1471-2148-8-143 (PMC2396631; doi:10.1186/1471-2148-8-143)
Supplement: Additional file 2 — Parameterization of the testicular architecture of the Drosophilide. [file 1471-2148-8-143-S2.rtf]

Additional file 2: Parameterization of the testicular architecture of the Drosophilide
General considerations
To establish an renewing epithelium requires two steps [1]. First, during embryogenesis and tissue formation cells divide in a exponential way to provide N stem cells to seed the tissue. During this exponential phase the cells may incur some level of somatic mutation as a function of the mutation rate per cell division during exponential division (ue) and the number of rounds of cell divisions required to make the stem cells (ne). This process leads to a population of N stem cells with a certain mutational load [2]. Second, established stem cells divide ns times via asymmetric mitosis, each time producing one stem cell and one transit cell. These divisions have a mutation rate of us per stem cell division. The resulting transit cells can then undergo nt symmetric mitotic divisions to form a clone of 2nt daughter cells (i.e. 4 cells if nt=2, 8 cells if nt=3, or 16 cells if nt=4, etc.), and mutating with a rate of ut per transit cell division. So each stem cell can make k differentiated cells either by varying ns, nt or both following the formula k=ns×2nt [3]. The complete tissue will thus consist of N stem cells that each produce k differentiated cells [1]. While the architecture of the exponential phase is fixed, there is scope for different architectures in the stem and transit cell phase.
One factor that has been suggested to influence the optimal topology is the mutation rate in stem cell divisions (us) vs. transit cell divisions (ut) [3]. If these mutation rates are the same, the optimal topology is the one that minimizes the number of divisions per cell, and thus the one that maximizes nt (i.e. produces the longest transit cell lineages). However, an epithelial organisation may constrain the length of the transit cell lineage (because transit cells are shed or lost from the tissue, and therefore can no longer divide) and thus '... stem cells are a necessary risk imposed by the constraints of the tissue architecture' [3]. In consequence selection would favour that stem cell divisions have a lower mutation rate than transit cell divisions (us<ut), in turn favouring a shift towards more stem cell divisions [3]. Testing the existing models of tissue architecture would thus require to obtain estimates of k, N, ne, ns, nt, ue, us and ut [1-3], and should therefore focus on species and tissues that allow to estimate at least some of these parameters. Although recent research suggests that us may be lower than ut in some tissues of some organisms [4, 5], there is little quantitative data on the absolute levels of mutation [4]. This lack of detailed knowledge could therefore make it difficult to test the models. A possible solution could be to make comparisons within species or between closely related species for which one could assume that the different mutation rates are (at least nearly) constant for a given tissue. In the following we assume that this assumption is approximately met for the testis of the Drosophilidae.
Specifics of the Drosophilidae
The testes of different species among the Drosophilidae are expected to vary greatly in N and/or k, and so they may require very different topologies within an otherwise fairly homogenous genetical background. Moreover, given the large variation in these parameters small variations in the mutation rates would probably not obscure the predictions. Moreover, in D. melanogaster detailed knowledge of the embryology and germ-line differentiation allows to estimate the number of cell divisions required to establish the testicular stem cell system, ne [6-8]. The primordial germ cells are differentiated at the 256 cell stage, and about N=16 of them are later incorporated into each testis as spermatogonia. Thus these germ-line stem cells experience about ne=8 cell divisions during the exponential phase. Moreover, there is a considerable understanding of the testicular stem cell system of D. melanogaster, such as the number of spermatogonial stem cells actually involved in spermatogenesis (N) which is somewhat stage-dependent [5] (N=15-16 in larvae and N=5-9 in adults), and which can be visualized and quantified using confocal laser scanning microscopy [6, 7]. SIt should be possible to obtain similar data for additional species. 
It has been estimated that a male D. melanogaster produces approx. T=150'000 sperm per testis over its lifetime [8]. However, D. Lindslay (per. comm.) considers this an overestimation. Tihen used an estimate of a sperm transfer rate from another study [9], which was based on males that had been sexually isolated for six days prior to mating, and which therefore probably had a backlog of mature sperm. D. Lindsley considers, that a more realistic number is about 12'000 sperm per male and week (pers. comm. and [10]), which, given an average life-span of about 35 days for male D. melanogaster [11], would yield about T=30'000 sperm per testis. Given these disparate numbers, better estimates would clearly be desirable. In the remainder we will use the lower estimate.
If we assume N=11 stem cells per testis (an average between the larval and adult values) this would require k=T/N=2727 sperm to be produced per individual stem cell. Lifetime spermatogenesis would then require ne=8 cell divisions during the exponential phase, followed by ns=43 stem cell divisions, each followed by nt=4 mitotic transit cell divisions and 2 meiotic divisions, i.e. k=43×4×24. So the first sperm to be produced would be 15 cell divisions from the original zygote, the last sperm 57 cell divisions from the original zygote, and an average sperm about 36 cell divisions from the original zygote [8]. Estimation of divergence at highly variable neutral loci, such as microsatellites, may allow to estimate to which degree mutations are more frequent in late that early sperm. Moreover, the number of stem cell divisions, ns, can probably be estimated directly with cell cycle studies using tritiated thymidine ([12]) or bromodeoxyuridine ([13]).
Several hundred species among the Drosophilidae are currently maintained in laboratory culture and can easily be obtained from resource centres for experimental work. So it should also be possible to obtain estimates of the life-time number of sperm produced by males (T=N×k). Assuming we can determine N this would therefore allow to estimate k. Whereas this number may be overestimated if determined under laboratory conditions (due to lower extrinsic mortality), it should still represent a useful approximation given the large amount of variation in this trait between species (likely more than three orders of magnitude). 
If there is proliferation-induced mutagenesis we would expect to find manifestations of testicular cancers or failed spermatogenesis in wild-type Drosophilidae. We are aware of no published data on this, but non-functional testes are not a rare observation when testes are dissected in the laboratory (D. Joly, pers. obs.). A more quantitative study, incoporating male age as a variable, would be highly interesting.
Finally, the high species richness and the increasingly well established molecular phylogenetic information (e.g. [17-20]) provide a rich source for future comparative analyses.
1.	Frank SA, Nowak MA: Problems of somatic mutation and cancer. Bioessays 2004, 26(3):291-299.
2.	Frank SA, Nowak MA: Developmental predisposition to cancer. Nature 2003, 422(6931):494-494.
3.	Frank SA, Iwasa Y, Nowak MA: Patterns of cell division and the risk of cancer. Genetics 2003, 163(4):1527-1532.
4.	Cairns J: Somatic stem cells and the kinetics of mutagenesis and carcinogenesis. Proc Natl Acad Sci USA 2002, 99(16):10567-10570.
5.	Hardy RW, Tokuyasu KT, Lindsley DL, Garavito M: Germinal proliferation center in the testis of Drosophila melanogaster. J Ultrastruct Res 1979, 69(2):180-190.
6.	Xie T, Kawase E, Kirilly D, Wong MD: Intimate relationships with their neighbors: tales of stem cells in Drosophila reproductive systems. Dev Dynam 2005, 232(3):775-790.
7.	Yamashita YM, Jones DL, Fuller MT: Orientation of asymmetric stem cell division by the APC tumor suppressor and centrosome. Science 2003, 301(5639):1547-1550.
8.	Tihen JA: An estimate of the number of cell generations preceding sperm formation in Drosophila melanogaster. Am Nat 1946, 80(792):389-393.
9.	Kaufmann BP, Demerec M: Utilization of sperm by the female Drosophila melanogaster. Am Nat 1942, 76:445–469.
10.	Lindsley DL, Tokuyasu KT: Spermatogenesis. In: The Genetics and Biology of Drosophila. Edited by Ashburner M, Wright TRF, vol. 2. London: Academic Press; 1980: 225-294.
11.	Min K-J, Tatar M: Drosophila diet restriction in practice: do flies consume fewer nutrients? Mech Ageing Dev 2006, 127:93-96.
12.	Chandley AC, Bateman AJ: Genetics: timing of spermatogenesis in Drosophila melanogaster using tritiated thymidine. Nature 1962, 193(4812):299-.
13.	Schärer L, Ladurner P, Rieger RM: Bigger testes do work more: experimental evidence that testis size reflects testicular cell proliferation activity in the marine invertebrate, the free-living flatworm Macrostomum sp. Behav Ecol Sociobiol 2004, 56:420-425.
